# The Effect of Occlusal Loading on Gingival Microleakage of Bulk Fill Composites Compared with a Conventional Composite

**DOI:** 10.30476/DENTJODS.2019.77861.0

**Published:** 2020-06

**Authors:** Razieh Hoseinifar, Maryam Mofidi, Nima Malekhosseini

**Affiliations:** 1 Oral and Dental Diseases Research Center, Dept. of Operative Dentistry, School of Dentistry, Kerman University of Medical Sciences, Kerman, Iran; 2 Dept. of Operative Dentistry, School of Dentistry, Kerman University of Medical Sciences, Kerman, Iran; 3 Dental Student, School of Dentistry, Kerman University of Medical Sciences, Kerman, Iran

**Keywords:** Composite Resin, Dental Leakage, Polymerization, Occlusal Loading

## Abstract

**Statement of the Problem:**

Bulk fill composites have been introduced over the recent years in order to accelerate the process of tooth restoration by inserting composite in bulk up to 4mm thickness. Occlusal loading may influence the gingival microleakage of this composite.

**Purpose:**

This *in vitro* study aims to evaluate the effect of occlusal loading on the gingival microleakage of bulk fill composites compared with a conventional composite.

**Materials and Method:**

In this experimental study, box only class II cavities with gingival margins placed 1mm below the cemento-enamel junction were prepared on the mesial and distal surfaces of 36 maxillary premolars (72 cavities). The samples were divided into three groups and restored as follows: Group 1 (Tetric N-Ceram, incremental filling), Group 2 (X-tra fill, bulk filling), Group 3 (Tetric N-Ceram Bulk Fill, bulk filling). All restorations were thermocycled for 2000 cycles (5-50̊C) and then half of the samples were subjected to 200,000 cycles of loading. All the specimens were immersed in 0.5% basic fuchsin for 48 hours, then, sectioned, and evaluated for microleakage with a stereomicroscope. Data were analyzed using Kruskal-Wallis and Mann-Whitney U-tests. *p*< 0.05 was considered significant.

**Results:**

There were no significant differences among the gingival microleakage of three composites in both unloaded and loaded groups. In addition, no statistically significant difference was found between the microleakage of unloaded and loaded groups in all materials.

**Conclusion:**

Occlusal loading did not affect the gingival microleakage of bulk fill composites, and the microleakage of class II cavities restored with the bulk filling technique was similar to that of restored with the incremental technique.

## Introduction

Although resin composites have been considered as the first choice for the direct restorations, their polymerization shrinkage has remained as a critical problem [ 1 - 2 ]. The volumetric shrinkage of composites is variable between 2% to 5%. The tensile stress of the composite shrinkage can cause debonding of the tooth-composite interface, which consequently, leads to postoperative sensitivity, enamel cracks, secondary caries, and microleakage [ 3 - 4 ].

Microleakage is one of the important factors, which affects the success rate of composite restorations and refers to the transfer of bacteria, liquids, and molecules between the prepared surface of tooth and restorative material [ 5 ]. It has been considered as a main challenge for the restorations of class II cavities with the direct composite, especially when the gingival margin is in the dentin [ 5 ]. The incremental technique is one of the methods for decreasing the polymerization shrinkage and increasing the marginal seal, but this method is time-consuming and there is a possibility of formation of bubbles among the restoration layers [ 1 - 2 , 6 ].

In order to accelerate the process of composite placement, new composites called bulk fill composites, have been introduced which can be inserted as a bulk to the depth of 4 mm according to the claim of their manufactures [ 7 ]. The main advantages of the bulk fill composites are their increased curing depth, which results from their higher translucency, and their lower polymerization shrinkage, due to the changes occurred in the content of filler such as the presence of iso-fillers or their resin matrix such as the presence of plasticizers or the polymerization modulator in the matrices [ 8 ].

Bulk fill composites have two consistencies; flowable and paste type. The flowable bulk fill composites are required to be covered with a final capping layer of the conventional composite, due to their low surface hardness and elastic modulus, but conventional bulk fill composites do not need this final coating [ 9 ]. 

The increased curing depth of bulk fill composites is due to their high level of translucency, the high volume of urethane dimethacrylate (UDMA) monomer, instead of bisphenole A glycidyl dimethacrylate (Bis-GMA) (UDMA indicated higher final degree of conversion than Bis-GMA). Moreover, it can be due to the presence of especial photoinitiator such as Ivocerin in Tetric N-Ceram Bulk Fill, the similar refractive indices of Bis-GMA monomer and Silica filler particles, the reduction in filler content, and increased dimension of filler particles (which decreases the matrix-filler interface. Thus, light scattering is decreased and allowing better light penetration) [ 10 ]. Bulk fill composites can be inserted into the depth of 4 mm in one layer. Therefore, working with them is easy and requires less time [ 11 ].

In the oral environment, restorations are under thermal and mechanical stresses and weakening of the adhesive resin caused these stresses are an important issue in operative dentistry [ 12 ]. In fact, the reaction of composite restorations to hydrolytic degradation and occlusal loading will define its resistance to fatigue and tooth-restoration interface breakdown [ 13 ]. Some studies indicated increased microleakage of composite restoration under the occlusal loading [ 12 , 14 ], while, the others reported that occlusal loading did not affect the marginal adaptation of composite restoration [ 15 - 16 ]. The aim of this study was to evaluate the effect of occlusal loading on the gingival microleakage of bulk fill composites (Tetric N-Ceram Bulk Fill and X-tra fill) compared to a conventional composite (Tetric N-Ceram), by the method of dye penetration and scanning electron microscopy (SEM) evaluation in class II cavities.

## Materials and Method

The samples of this experimental *in vitro* study consisted of 36 extracted maxillary premolar teeth. They were extracted for orthodontic treatments and had intact surfaces, without caries and decalcification. The teeth were disinfected in the 0.5% Chloramine-T solution for one week and then, were kept in the normal saline solution. 

### Cavities preparation

A total of 72 standard cavities of class II (box only) with the buccolingual width of 4mm, the depth of 1.5 mm and the occlusal-gingival length of 1 mm under cemento-enamel junction were prepared on the mesial and distal surfaces of all teeth, using a water-cooled high speed hand-piece and the fissure diamond bur (Tizkavan, Tehran, Iran). By cutting five cavities, the bur was changed. The materials used in the present study and their chemical compositions were mentioned in Table 1. First, the metal matrix band was fixed using a Tofflemire holder. Then, all the cavities were etched with 37% phosphoric acid gel (Total Etch, Ivoclar Vivadent) for 15 seconds, washed thoroughly, and the excess moisture of each cavity was removed with a small cotton pellet. Subsequently, two layers of Tetric N-Bond (Ivoclar Vivadent) were applied 10 seconds by micro brush on the walls of cavities, gently air dried, then light cured for 20 seconds with a light emitting diode (LED) curing unit (DEMI, Kerr, USA) at 800 mW/cm² intensity. Afterward, the samples were divided into one of the following groups randomly.

**Table 1:** The materials used in this study and their composition

Material	Composition	Manufacturer	Batch number
Tetric N-Bond	Phosphoric acid acrylate, HEMA, Bis-GMA, UDMA, ethanol, film-forming agent, catalysts, and stabilizers	Ivoclar Vivadent, Schann, Liechtenstein	V37028
X-tra fill	Bis-GMA, UDMA, TEGDMA, Fillers: 86% wt, 70% vol, Ba-B-Al-Si glass	Voco Cuxhaven, Germany	1633494
Tetric N-Ceram	UDMA, ethoxylated Bis-EMA, Bis-GMA (18.8 wt%), barium glass filler, ytterbium trifluoride, mixed oxide (63.5 wt%), polymer (17.0 wt%), additives, catalysts, stabilizers, and pigments (0.7 wt%)	Ivoclar Vivadent, Schann, Liechtenstein	V23282
Tetric N-Ceram Bulk Fill	Dimethacrylates 21.0% (Bis-GMA, Bis-EMA, UDMA) Polymer Filler 17.0% (Barium glass filler, Ytterbium trifluoride) Mixed oxide 61.0% Additive, Initiators, Stabilizers, pigments, 1.0%	Ivoclar Vivadent, Schann, Liechtenstein	V19409

HEMA: 2-hydroxyethyl methacrylate, UDMA: urethane dimethacrylate, Bis-GMA: bisphenol A glycidyl dimethacrylate, TEGDMA: triethylene glycol dimethacrylate, Bis EMA: ethoxylated bisphenol A glycol dimethacrylate.

In the group 1, the cavities were restored with Tetric N-Ceram composite (Ivoclar Vivadent) incrementally (with 2mm thickness in each layer) and each layer was cured for 20 seconds.

In the group 2, the cavities were restored with X-tra fill composite (Voco, Germany) as bulk (a 4mm thick increment was placed into the cavity and cured for 20 seconds, followed by the next increment to entirely fill the cavity and cured for 20 seconds). 

In the group 3, the cavities were restored with Tetric N-Ceram Bulk Fill (Ivoclar Vivadent) as bulk (a 4mm_thick increment was placed into the cavity and cured for 20 seconds, followed by the next increment to fill the cavity entirely and cured for 20 seconds).

**Figure 1:**
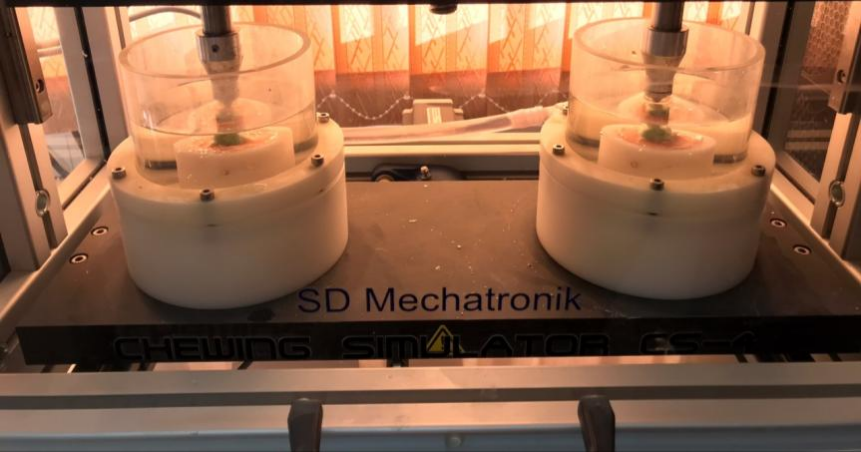
Studied specimens in chewing simulator machine

In all groups, after removing the matrix strip, the restorations were cured from the buccal and palatal aspects for 20 seconds on each side, and then all the restorations were finished and polished by diamond finishing burs and polishing disks (Soflex, 3M, ESPE, USA). After keeping them in an incubator at 370C for 24 hours, the samples were subjected to 2000 thermal cycles in water bath between 5-500C (dwell time: 30 seconds in every bath and transfer time: 20 seconds) (Baradaran Pouya, Iran). Then in each group, half of the samples were kept in an incubator at 37 0C and the other half of the samples were mounted up to 1mm apical to cervical margins of restorations in self-curing acrylic resin (Acropars, Iran). Then they were subjected to 200,000 cycles of loading with a force of 60 N, frequency of 2 He-rtz and displacement of 1mm by using a chewing simulator machine (Germany, SD Mekantronik) (Figure 1).

### SEM evaluation

**Figure 2:**
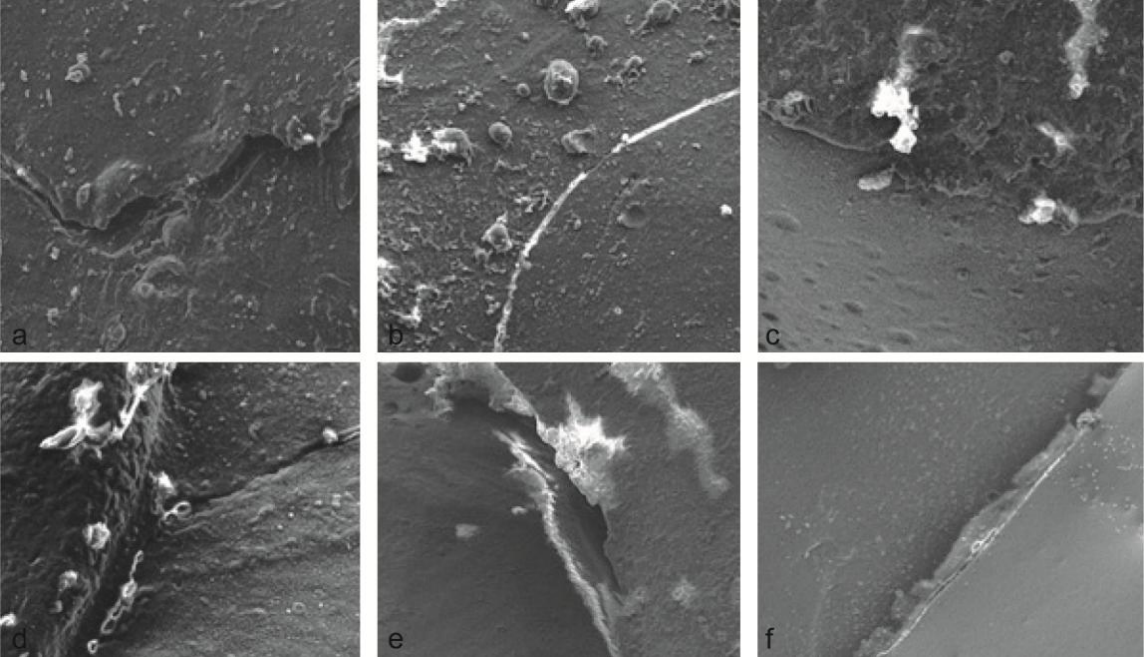
SEM micrograph of tooth – composite interface of unloaded groups (X-tra fill (a), Tetric N- Ceram Bulk fill (b), Tetric N- Ceram (c)) and loaded groups (X-tra fill (d), Tetric N- Ceram Bulk fill (e), Tetric N- Ceram (f))

Before sectioning the samples, an impression (precise, Coltene, Switzerland) was taken off the surface of 12 restorations, two samples in each subgroup, and the positive epoxy resin replica was gained from each sample. Each replica was placed on a metallic stub and sputter coated with a thin layer of gold and was evaluated with a SEM microscope (TESCAN-Vega3, Czech Republic) with 1000X magnification. Then, interfacial gaps were measured (Figure 2). The whole length of all gaps was shown as a percentage of all lengths of the restoration margins. 

### Microleakage evaluation

**Figure 3:**
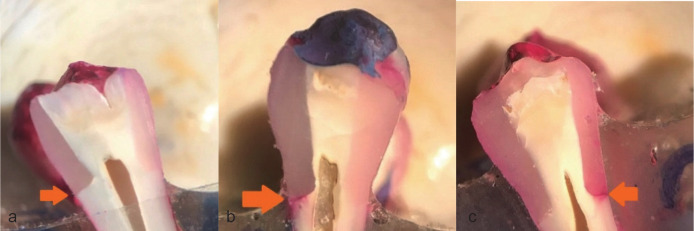
The picture of sectioned samples under stereomicroscope, a; no dye penetration, b; dye penetration more than ½ of the gingival wall, which does not reach the axial wall, c;dye penetration along the axial wall

All surfaces of the teeth except the parts that have been filled and 1 mm around the margins were sealed with two layers of nail polish. Then, the teeth were immersed in the 0.5% basic fuchsin solution for 48 hours. Samples were washed with distilled water, dried, and embedded in self-curing acrylic resin. In the next step, the teeth were sectioned longitudinally in the mesio-distal direction through the center of restorations using a cutting machine with low- speed diamond disk (Presi, Mecantome, T201A, France) under continuous water irrigation. After that, the samples were assessed using a stereomicroscope (Nikon, 30DS, SMZ 800, Tokyo, Japan) with a magnification of 40X (Figure 3). The degree of dye penetration was scored as (0) for absence of dye penetration, (1) for dye penetration up to 1/2 of the gingival wall, (2) when dye penetration was more than 1/2 of the gingival wall but does not reach the axial wall, and (3) when dye penetration was present along the axial wall.

The statistical analyses were done by using Kruskal-Wallis and Mann-Whitney U tests. The p Value of <0.05 was considered as the significant level.

## Results

**Figure 4:**
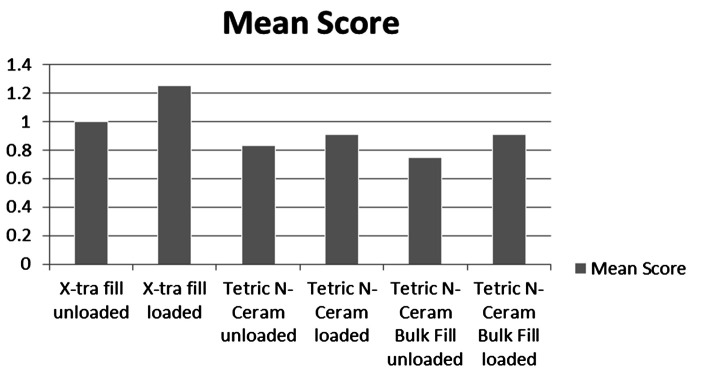
The mean of microleakage score for the studied composites.

The results of the gingival microleakage of restored samples with different composites are shown in Table 2 and Figure 4. There were no significant differences among the gingival microleakage of three composites in both unloaded and loaded groups (*p* > 0.05). Moreover, there was no significant difference between the bulk filling and incremental filling technique. Furthermore, no statistically significant difference was found between the gingival microleakage of unloaded and loaded groups in all composites (*p*> 0.05). Table 3 shows the inter-facial gaps observed under SEM.

**Table 2:** The results of gingival microleakage of tested composites

Groups		Microleakage			
		Score 0	Score 1	Score 2	Score 3
X-tra fill	Unloaded	3	6	3	0
	Loaded	1	8	2	1
Tetric N-Ceram	Unloaded	4	6	2	0
	Loaded	3	7	2	0
Tetric N-Ceram Bulk Fill	Unloaded	5	5	2	0
	Loaded	4	6	1	1

**Table 3:** The results of interfacial gaps observed by SEM

Filling Materials	Loading Status	The mean percentage of interfacial gaps of two specimens of each group (%)
X-tra fill	Unloaded	1.25
	Loaded	1.67
Tetric N-Ceram	Unloaded	0.92
	Loaded	1.17
Tetric N-Ceram Bulk Fill	Unloaded	1.06
	Loaded	1.81

## Discussion

The important factor, which determines the preservation of composite restoration is the marginal seal and lack of leakage [ 7 ]. 

The present study indicated that there were no significant differences among the gingival microleakage of three kinds of composites in both loaded and unloaded groups. In addition, there was no significant difference between bulk filling and incremental filling method, which is in agreement with the findings of some studies [ 17 - 18 ]. Heintze *et al*. [ 17 ] evaluated the quality of gingival margins of class II composite restorations, which placed in bulk or three increments, and showed that there was no significant difference between two techniques. Furness *et al*. [ 18 ] restored the cavities of class I with bulk fill (SDR, Quixx, Sonic Fill, Tetric EvoCeram) and a conventional composite, Filtek Supreme, and indicated that there was no significant difference between the marginal integrity of two composite placement methods. 

Kim *et al*. [ 19 ] found that the flowable bulk fill composites indicated better performance in terms of composite-tooth interfacial de-bonding than the conventional flowable composites, due to their lower polymerization shrinkage and elastic modulus. In the bulk fill composites, their lower polymerization shrinkage and elastic modulus decreased the amount of microleakage [ 20 ]. It is believed that the occlusal loads and the thermal changes lead to the gap in the interface of teeth-restoration [ 21 - 22 ]. In this study, 200000 cycles of loading were applied to mimic one year of service in vivo [ 23 ]. The results of the present study showed that the microleakage of gingival margins of three composites was not affected by cyclic loading. This finding is consistent with the previous studies [ 15 - 16 ]; However, some studies indicated the increase of microleakage of composite restorations under the cyclic loading [ 14 , 24 ].

Campos *et al*. [ 25 ] evaluated the marginal integrity of bulk fill composites (Surefill SDR, Sonic Fill, Venus Bulk Fill, and Tetric Evo-Ceram) in the class II cavities after 240000 cycles of loading and showed that occlusal loading did not affect the marginal adaptation of bulk composites and only Venus composite showed increased gingival microleakage after loading.

Shahidi *et al*. [ 24 ] evaluated the effect of 1,000,000 cycles of loading on marginal adaptation of class II cavities restored with Surefil SDR, Ceram X, Sonic Fill, Tetric, and Extra-low shrinkage composites. They reported that the effect of occlusal loading on the gingival marginal adaptation of all groups except for Tetric was statistically significant, which was not in agreement with the current study results. Jung *et al*. [ 14 ] reported that after applying the 600,000 cycles of loading, the microleakage was significantly increased in the gingival margins of restored class II cavities by bulk fill composites (SDR, Sonic Fill, Venus bulk fill, Tetric N-Ceram Bulk Fill), which is not in accordance with the result of this study. Such different results may be explained by the differences in the number of cycles of loading (1,000,000 and 600,000 instead of 200,000). The higher number of cycles may have a more destructive and damaging effect on marginal adaptation [ 24 ]. The gap formation in composite restorations is the result of different parameters such as the restorative materials stiffness, the degree of conversion and the polymerization shrinkage of composites [ 26 ]. The sufficient cure and degree of conversion of composites is one of the criteria, which affect the marginal adaptation, and inadequate polymerization of composites might lead to marginal microleakage [ 27 ]. The previous studies have reported the sufficient degree of conversion of X-tra fill and Tetric N-Ceram composites at the depth of 4mm [ 14 , 27 ]. In a study conducted by Abed *et al*. [ 27 ], X-tra fill showed significantly the highest degree of conversion. Jung *et al*. [ 14 ] also reported the higher bottom/top surface hardness ratio of Tetric N-Ceram Bulk Fill (82%), than the other evaluated composites, which is due to the presence of the special initiator (Ivocerin). It is a germanium-based photoinitiation and has a higher absorption spectrum compare to camphorquinone [ 14 ]. The sealing ability of restorative materials also depends on the type of material and adhesive systems [ 28 ]. It has been shown that the properties of resin composite affect the resistance to marginal degradation more than the marginal adhesion [ 29 ]. Considering the role of composite, elastic modulus and the amount of polymerization shrinkage are the main factors affect the marginal integrity of composite restorations [ 30 ].

Low shrinkage composites provide lower shrinkage stresses during curing. Thus, they are able to withstand fatigue at the tooth-restoration interface better than the other resin composites [ 3 ]. Kleverlan *et al*. [ 31 ] reported a strong linear correlation between polymerization shrinkage stress and gap formation. Jung *et al*. [ 14 ] also indicated that after loading correlation between marginal integrity and linear polymerization shrinkage was higher than preloading, which is due to the weakness of the bonding between tooth and composite through the loading process. Therefore, better adaptation would result in lower polymerization shrinkage [ 14 ]. 

Bulk fill composites show less polymerization shrinkage, due to the use of stress-reducing resin technology. This technology is based on the changes in the chemistry of monomers [ 32 ]. Manufacture companies changed the Bis-GMA monomer, which resulted in the production of monomers with lower viscosity such as Bis-GMA without the hydroxyl group, aliphatic urethane dimethacrylate, partially aromatic UDMA, and methacrylate with several branches. These changes decreased the polymerization shrinkage of bulk fill composites [ 32 ]. Likewise, in the bulk fill composites, the reaction of polymerization occurs more slowly, which decreases the shrinkage stress without compromising the degree of conversion [ 30 ]. 

The other possible explanation refers to the use of composites with nanofiller content in this study (Tetric N-Ceram Bulk and Tetric N-Ceram). Cyclic forces decrease the performance of bonding, due to fatigue at the adhesive interface. Some investigations indicated that nano-composites had a higher fatigue limit, due to their 

higher compressive strength [ 33 - 35 ]. 

## Conclusion

Based on the limitations of this study, occlusal loading did not increase the gingival microleakage of bulk fill composites. In addition, the microleakage of class II cavities restored with the bulk filling technique was similar to that of restored with incremental technique.
